# Broken drug markets in infectious diseases: Opportunities outside the private sector?

**DOI:** 10.1371/journal.pntd.0007190

**Published:** 2019-04-11

**Authors:** Jonathan D. Alpern, Stephen J. Dunlop, William M. Stauffer

**Affiliations:** 1 Division of Infectious Diseases, Department of Medicine, HealthPartners, Minneapolis, Minnesota, United States of America; 2 HealthPartners Institute, Minneapolis, Minnesota, United States of America; 3 Department of Emergency Medicine, Hennepin County Medical Center, Minneapolis, Minnesota, United States of America; 4 Department of Medicine, University of Minnesota-Minneapolis, Minnesota, United States of America; Saudi Ministry of Health, SAUDI ARABIA

## Abstract

A subset of anti-infective drugs are increasingly unavailable for patients in the United States due to pricing or withdrawal from the market. Timely market solutions are needed. We assert that solutions to ensure access to some essential anti-infective agents lie outside capital markets and that public-private partnerships may be the most viable solution.

Substantial price hikes of off-patent pharmaceutical drugs have become common. In response, a variety of policy solutions have been proposed to curb exploitative price increases for off-patent drugs. For example, the US Food and Drug Administration (FDA) now expedites generic manufacturer applications for drugs in noncompetitive markets and recently published a list of off-patent, sole-source drugs to encourage generic competition [1]. In addition, the state of Maryland became the first state to pass anti-price gouging legislation, and other states are considering similar legislation [2]. Recently, the US Senate Judiciary Committee advanced a bipartisan bill known as the “Creating and Restoring Equal Access to Equivalent Samples” (CREATES) Act, aiming to thwart anticompetitive practices by brand name manufacturers and to promote competition among generic manufacturers [3].

While policies aimed at encouraging generic manufacturer competition or outlawing price gauging may be effective for keeping prices reasonable for high-volume drugs (i.e., EpiPen for anaphylaxis), these solutions will likely be ineffective for drugs in low demand in the US, including many important anti-infective drugs. Many of these drugs have characteristics that make the market in the US nonviable: off-patent, cheap to manufacture, very limited volume for potential sales, and frequently used for neglected infections that affect low-income populations.

Pharmaceutical companies that sell these drugs are faced with difficult choices: continue to sell at minimal profit (or a loss), withdraw from the market, raise prices, or sell the license to a third party. Current policy solutions and public attention has been directed at pharmaceutical companies cornering the market for an essential drug and charging “what the market will bear” [4]. We are concerned that, in the case of well-intentioned drug price gouging legislation, there may be unintended consequences that threaten the availability of these important drugs. Forced to charge reasonable prices, companies may discontinue manufacturing of drugs with a small market share and limited, or no, profit potential. For drugs with a single manufacturer, this could lead to a drug’s withdrawal from the market all together.

Consider the monopolized antiparasitic drug market that has exhibited steep price increases recently [5]. Prior to the popularity of this business model, few manufacturers were attracted to this space due to limited profitability, and drug discontinuation from the US market was common. Some of these withdrawn drugs remain commercially unavailable, such as quinacrine (giardia), niclosamide (tapeworm), and diethylcarbamazine (filarial diseases) [6]. More recently, in December 2017, the sole manufacturer of intravenous quinidine gluconate discontinued manufacturing for reasons not related to safety or efficacy, with plans to continue product distribution only through March 1, 2019 [7]. Quinidine is the only FDA-approved intravenous drug available for the treatment of severe malaria in the US. While the reasons for discontinuation have not been publically disclosed, limited profitability due to low demand in the US is likely the primary factor. Historically, its primary use in the US has been treating atrial and ventricular arrhythmias, not malaria, and use has decreased with the adoption of safer antiarrhythmic drugs. Since US providers and hospitals will no longer have access to parenteral quinidine, parenteral artesunate will need to be acquired from the Centers for Disease Control (CDC). Although artesunate is considered first-line therapy for severe falciparum malaria, the drug is not currently FDA approved in the US and is available solely through the CDC as an investigational new drug (IND). Access to artesunate requires obtaining permission from the CDC, shipment from one of quarantine station repositories, obtaining emergent approval from an Institutional Review Board and patient consent, resulting in delays in administration. The FDA approval of artesunate would be a welcome development, ensuring improved access to treatment for a disease that can be fatal in hours. However, with quinidine now unavailable—even in the best circumstances—there will be delays in therapy for patients with severe malaria infection until there is an FDA approved drug available in the U.S that is standard of care for treatment of severe malaria.

The fragility of high-value but low-volume drugs is perhaps best exemplified by the arsenal of drugs for multidrug-resistant tuberculosis (MDR-TB). Of the first- and second-line drugs indicated for the treatment of MDR-TB (*n* = 12), over one-half have two or fewer manufacturers, and five of these drugs are produced by only one manufacturer (Table 1). While these drugs are at risk for exploitative pricing strategies—as occurred briefly with Seromycin (cycloserine)—the lack of generic competition in this space and the small market suggests that drug discontinuation and withdrawal from the market is another risk.

**Table 1:** First- and second-line MDR-TB drugs.

Drug	Number of manufacturers
Pyrazinamide 500-mg tablet*	2
Myambutol (ethambutol hydrochloride) 400 mg*	4
Levaquin (levofloxacin) 250-mg tablet	15
Avelox (moxifloxacin hydrochloride) 400-mg tablet	9
Amikin (amikacin sulfate) 250-mg/ml injection	5
Streptomycin (streptomycin sulfate) 1 gram/vial injection	1
Kanamycin (kanamycin sulfate) 500-mg/2-ml injection*	1
Capastat sulfate (capreomycin sulfate) 1 gram/vial injection*	2
Seromycin (cycloserine) 250-mg capsule*	1
Trecator (ethionamide) 250-mg tablet*	1
PASER (aminosalicylic acid) delayed-release granule 4 gram/packet*	1
Zyvox (linezolid) 600-mg oral tablet	11

**Abbreviations:** FDA, Food and Drug Administration; MDR-TB, multidrug resistant tuberculosis.

*Drugs with TB as the sole FDA indication.

Data on the number of manufacturers is from the FDA Orange Book.

Drug names accessed from Fig 2 in “Drug-Resistant Tuberculosis: A Survival Guide for Clinicians,” Curry International Tuberculosis Center [8].

When important drugs are withdrawn from the US market for reasons other than safety or efficacy, there are limited mechanisms to ensure they remain available to patients that need them. The CDC drug service currently stocks 12 biologic agents and drugs considered essential for public health that are not available through commercial avenues [9]. Many of these drugs are obtained through foreign sources and lack FDA approval, requiring the CDC to sponsor an IND in order to legally distribute them in the US. Although the CDC formulary is an important resource for individual patients, relying on this drug service as a “safety net” for essential drugs—if more are withdrawn—is tenuous. Multiple barriers exist, including the lack of a mechanism for drug distribution (currently done on an individual patient basis) and timeliness for severe diseases such as malaria.

Meanwhile, for-profit pharmaceutical companies remain unreliable sources for drugs that are essential to human health but have limited profit-generating potential. For drugs with these characteristics, the market is broken because companies either corner the market and charge “what the market will bear,” or sell the drug at a reasonable price, which limits profitability. In the latter example, withdrawal from the market becomes a risk, as occurred with intravenous quinidine. Therefore, relying on the free market for a rational solution seems futile and other policy solutions are needed.

One approach to ensure the availability of drugs in these broken markets is to establish a public–private partnership between the federal government and candidate drug manufacturers. Such a relationship exists with the Vaccines for Children Program (VFC). Passed in 1993, the VFC provides vaccines to vulnerable children who would otherwise not be able to afford the vaccine. In this program, the CDC purchases discounted vaccines recommended by the Advisory Committee on Immunization Practices from vaccine manufacturers and distributes them to VFC grantees, such as state health departments and other local health agencies. The vaccines are then distributed at no cost to VFC registered clinics [10]. Yet, the VFC model would be difficult to replicate since sales volumes for vaccines are significantly higher than they would be for low-volume drugs, limiting profitability for drug manufacturers in such a program.

On the contrary, existing infrastructure within the federal government could be leveraged to ensure availability through a mechanism like the Strategic National Stockpile (SNS). The SNS is managed by the Department of Defense and FDA and maintains a large formulary of critical, life-saving medical products. Through partnerships with the public and private sector, the SNS is able to ensure the timely delivery of large volumes of product [11]. To incorporate broken market drugs into the SNS, the federal government could purchase candidate drugs at risk of being discontinued from the US market from the drug manufacturer. Certain drug characteristics could be defined for inclusion on this list, such as FDA-approved drugs that are considered essential by WHO, lack readily available therapeutic alternative options, are in low demand in the US, and are produced by one manufacturer.

Such a model is not without precedent. Drawing from experience in the agricultural sector, the government has supported US farmers through subsidies for years to ensure stable pricing and supply. In the case of the dairy sector, government subsidies totaled approximately US$22.2 billion dollars in 2015. Federal funds ensure that dairy farmers’ cost of production does not exceed profits from sales in the market [12]. Similarly, the funds received by drug companies from the federal government for the purchase of candidate drugs would ensure a guaranteed purchase order such that the costs of production do not exceed sales. Maintaining the inventory of broken market drugs would require oversight already in place within the SNS and would not require restructuring of the program. However, timely delivery of drugs to patients requiring them would be the primary barrier to this model since the SNS is currently structured to deliver large volumes of products primarily in the setting of public health emergencies.

Alternatively, the government could allow the FDA to approve drugs manufactured outside of the US based on evidence already considered and approved by trusted international regulatory bodies, such as the European Medicines Agency and Health Canada [13]. Although multiple US states have issued bills to allow drug importation from Canada, this policy solution would not have an impact on drugs that are not available in Canada, which includes many of the antiparasitic drugs.

Many hurdles exist to implementing this novel, public–private partnership approach to drug access in the US. A rigorous process for defining inclusion criteria will be needed in an arena with little precedent. For instance, determining the value of drugs in the US has been problematic [14]. In addition, other factors such as public health importance and vulnerability of a drug’s target population would also need to be considered in addition to a drug’s status as an essential medicine. Although “access to life-saving drugs” is a priority to the current administration [15], the overall cost of such a program may not be feasible considering recent budgetary cuts. Finally, efficient and timely distribution channels would be a crucial component of the program, especially considering that many anti-infective drugs require early administration.

In concert with efforts aimed at decreasing off-patent drug costs, we must also consider the impact that such efforts could have on the availability of life-saving drugs. While we are encouraged that some state legislators are making attempts to remedy high drug prices, if price gouging legislation is broadly adopted, this may worsen availability of certain essential drugs. Acknowledgment that this sector of the market is broken is a necessary first step and should be followed by timely policies to ensure these drugs remain available in the US.

To do this effectively, first, we propose that state lawmakers creating legislation aimed to address price gouging for essential medicines collaborate with key representatives from the pharmaceutical industry. While pharmaceutical companies currently engaged in exploitative pricing strategies are unlikely to provide objective feedback, drug companies that have already discontinued an essential drug for reasons not related to safety or efficacy are important resources. Second, federal or state lawmakers should explore the possibility of utilizing the SNS as a tool to ensuring availability of off-patent essential drugs. This could be implemented in a scalable way by starting with legislation in one state and, if successful, scaling more broadly to other states or at the federal level.

Until policy solutions are developed, the discontinuation of quinidine—the only FDA-approved intravenous treatment for severe malaria—should serve as a sobering reminder of what is at stake if these issues are not addressed.
